# Treatment of Dental Caries, Complicated with Disorders of the Pulp and Peridental Membrane

**Published:** 1852-07

**Authors:** Robert Arthur


AMERICAN JOURNAL

OF

DENTAL SCIENCE.

Vol. II. NEW SERIES- JULY, , 1852. NO. 4.

### ARTICLE I.

*Treatment of Dental Caries, complicated with Disorders of the Pulp and Peridental Membrane.* By Robert Arthur, D. D. S., M. D.

### NO. V.

In the last number I endeavored to show the propriety and utility in some cases, and the necessity in others, of the extirpation of the pulp when it becomes exposed. I also explained the object expected to be accomplished by the performance of this operation, and discussed the question of the relative propriety of using arsenic for the purpose of destroying the vitality of the pulp previous to its removal, and taking it away without preparatory treatment. I endeavored to show that the objections commonly made to the use of arsenic for this purpose were not well founded.

The indications which are to determine the necessity of a resort to this operation, the best manner of using arsenic for the purpose, the symptoms developed by its action, and the manner of removing the pulp, with other relative subjects, will occupy the present number.

I have insisted strongly upon the importance of preserving the pulp alive and in healthy condition, when this can be done, but in many cases it will be found exceedingly difficult to determine whether the entire extirpation of the pulp is absolutely necessary, or whether the means indicated in a former number are applicable; whether the caries has reached so nearly to the pulp as to render useless or injurious any attempts to fill over it.

It is my decided impression, although I have not yet perfectly satisfied myself that it is a fact—that when the pulp is once fully reached by the caries, that it is generally beyond treatment except by extirpation. I believe that contact with the decomposed bone and the chemical agents which bring about its decomposition, will invariably and inevitably produce inflammation of the pulp; and I am inclined to believe that where the pulp remains in a perfectly healthy condition, covered by softened bone, that this is not decomposed, but as I have already intimated, bone in the first stages of formation; and that in all cases where the pulp is carefully uncovered so as to perform the operation of "capping," as it is technically termed, before pain has occurred, that a perfectly healthy substance is taken away, and that the very questionable alternative is taken of bringing and keeping the pulp in contact with atmospheric air. That under such circumstances, a new protective covering of bone may be secreted by the pulp I cannot deny, as it has been positively affirmed by gentlemen in the profession, of high standing, and of whose skill and truthfulness there cannot be a doubt, but I am obliged to say, that no fact which satisfies me of its truth has ever come within my own observation.

All cases, then, in which no pain of any kind, or other evidences of inflammation has occurred, no matter what may be the extent of the caries, I regard as within the reach of the operation indicated in a former number and alluded to above. I state this as a general rule, for I am aware that many persons lose their teeth by decay, without, according to their own statement, suffering pain; but I doubt whether such teeth did not give rise in the progress of the destruction of the pulp to some painful symptoms of inflammation, although they may have been very slight.

Pain, therefore, in my view of the case, may be regarded as an indication of such a condition of the pulp of a tooth as to call for its removal. But as pain occurring in and about the teeth may come from a variety of causes, it must be understood that I mean pain occurring under certain circumstances, and of a peculiar character.

In order to make this subject clear, it will be necessary to go briefly into a consideration of the various kinds of painful symptoms of the teeth, and their causes, to distinguish them from that which occurs in consequence of true inflammation of the pulp. This has already been very clearly done by Dr. White, in the series of articles alluded to in the introductory number of these papers, and although what I have to say may not differ materially from what will be found in them, it is necessary in this connection, and must, of course, take some peculiar coloring from my own view of the matter.

I shall notice, amongst the causes which give rise to painful sensations about the teeth,

1st. Inflammation of the dentine.

2nd. Inflammation of the peridental and alveolar membranes.

3rd. Sympathy with other painful teeth.

4th. Neuralgia.

5th. True inflammation of the dental pulp from contact with substances foreign to it.

1st. Pain caused by inflammation of the dentine may occur at any stage of the progress of caries, and frequently does occur when it is very slight. It is rarely intense, and is sometimes characterised by patients as a "grumbling" pain. Sometimes it can scarcely be called pain, a simple consciousness of something wrong, an uneasiness about the part, is all which indicates its presence. In ordinary states of health, pain rarely occurs spontaneously in such cases, but cold—an extraordinarily plethoric state of the system—febrile excitement—

pregnancy—inflammation of the pulp of some other tooth on the same side of the mouth, are sufficient to induce it. Contact with any hard, and sometimes with moderately hard, substances, or substances below or above the temperature of the blood, is sufficient at times to cause pain, and an attempt to remove the dentine when it is in this condition, with a cutting instrument, will occasion, to some persons, pain of a peculiarly excruciating character. I have already alluded to this in a former number.

2nd. Inflammation of the membranes of the alveolar sockets. From this cause, very acute and long continued pain, which, from its character, it is difficult to distinguish from pain caused by a high degree of inflammation of the pulp, may be experienced.

In these cases there is always more or less tenderness to pressure, which, indeed, will always determine whether there is any inflammation of the peridental membrane, or of that which lines the alveolus. The pain may vary in a degree from slight uneasiness about the part to exceedingly acute toothache, as it is commonly termed.

The diagnosis of these cases, however, is not very difficult, for it is generally occasioned by teeth in which the nerves are dead. It may, it is true, and frequently is, complicated with inflammation of the pulp arising from any cause.

It is also occasioned by tartar encroaching upon the fangs and destroying as it accumulates, the gum, alveolar processes, and the membranes of the sockets. Some of the most severe cases I have ever met with owe their origin to this cause.

In these cases the contact of cold or hot substances will sometimes occasion severe pain. Sometimes, too, it may be well to mention, while on this subject, severe pain follows the extraction of teeth in this condition, continuing with great violence for several days after their removal.

3rd. Sympathy with other painful teeth. It is a fact well known to those who have observed the phenomena attendant upon diseased teeth, that pain may be felt in those which are quite distant from the tooth, or teeth, in which it originates. This is so common, that an experienced dentist will never rely entirely upon the patient's indication of the painful tooth when extraction is required. Mere proximity does not account for the fact, as sometimes teeth in the opposite jaw to the one which may be affected will be painful. Sometimes the really diseased tooth will not be at all painful, and will only be detected by the fact that it is found to be carious, and to some extent, affected with peridental inflammation. This kind of pain may occur in a tooth perfectly sound; although I am not so well satisfied that this is so; but it is generally an indication that the tooth in which the pain exhibits itself from sympathy with one which is diseased, is, itself, more or less diseased, and attention ought to be directed to it, after the one which is the original cause of the painful symptoms is attended to.

Pain occurring from this cause varies in intensity; it may be slight, and the whole of the teeth on one side of one of the jaws or face be affected at the same time, or it may be confined to a single tooth, and thus may exhibit all the characteristics of pain arising from genuine inflammation of the pulp.

4th. Neuralgia.—This is another prolific source of painful sensations about the teeth, and this may affect teeth which are perfectly sound, and apparently healthy. This affection, obscure in its pathology, and exceedingly difficult of treatment, is one, to the observation and study of which, every dentist ought to give careful attention.

The character of the pain of neuralgia varies so much that it cannot be described unless all the terms used to describe painful sensations are employed. A general distinction, however, between pain occurring from this and other causes, has been made, which will assist, to some extent, in judging of the presence of neuralgia. "The description of pain, unattended with inflammation, differs from the pain of inflammation, although the former is subject also to varieties in kind, duration, and intensity. Throbbing, lancinating, or pulsatile pain, *i. e.*, pain accompanied with a sense of motion of the fluids in the parts, is the most characteristic distinction of acute inflammation; and an obtuse aching, or heavy pain, belongs to a congested state of the local circulation. Neuralgia is generally attended, more or less, with muscular cramp or spasm, and such pain is either intermitting or periodical.—*Travers* on *Inflammation*, p. 47.

I am not sure that the condition of the affected teeth materially modifies this affection. As far as my experience goes it is quite as liable to exhibit itself in perfectly sound teeth as in any others. Where there are no diseased teeth in the mouth, and pain occurring, cannot be attributed to any of the causes enumerated above, and about to be stated, it may be suspected to proceed from this cause, and an effort made to account for it on this ground.

5th. True inflammation or congestion of the dental pulp.—I come now to the consideration of the circumstances under which pain occurring, may be suspected to proceed from this cause.

Pain produced by inflammation of the dental pulp is not so peculiar in its character as to be at once, and in all cases, distinguished from pain arising from any one of the causes just enumerated. In many cases it may so nearly resemble any one of the species here alluded to, that it cannot be distinguished from it. This is what renders diagnosis in the case sometimes difficult.

Sometimes, however, the pain resulting from inflammation of the pulp, together with the extent to which the painful tooth is affected with caries, is of such character that it cannot be mistaken, and no operator of any experience will be deceived. But as the inflammation or congestion giving origin to painful symptoms may subside spontaneously without leaving a trace behind, and the patient forgetting what he had suffered, does not describe accurately the symptoms, and as it can never be ascertained with perfect precision, by examining the cavity of decay, how nearly the caries has approached the vicinity of the pulp, it frequently becomes an exceedingly nice question to determine, whether an attempt may, with any hope of success, be made to preserve the pulp alive, or whether the operation of which I am now treating is indicated. There are some instances, indeed, in which this is actually impossible, and the matter can only be determined by resorting to a test which I shall presently describe.

On examining the teeth of a patient in my hands, if I find any which, from the depth, extent, and situation of the caries, give me reason to believe that the pulp has suffered, or is in danger of suffering, I proceed to ascertain their exact condition.

I first inquire of the patient if he has suffered pain from the tooth, or teeth, in question. If none has occurred, and strong, hard pressure made upon a pledget of cotton placed in the cavity gives no pain, I proceed as in ordinary cases.

If no pain has occurred previously, and strong pressure produces pain, which, however, immediately passes away, I proceed as already directed in the third number of these papers.

It sometime happens, even before the pulp is in any way injuriously affected, and the practice above is applicable, that pressure made, as directed here, will produce pain which does not immediately subside. In many of these cases, the teeth will be insensible to the effects of external agents, with which they may ordinarily be brought into contact, and as a general rule, they may be distinguished by the fact that no pain of consequence has, up to the time of examination, occurred spontaneously; or if pain may have occurred, it is of such character as could be referred to inflammation of the dentine.

If violent and continued tooth-ache has occurred, although the tooth coming under treatment may be perfectly free from pain at the time, it may be generally regarded as an indication that the pulp is in such condition that it cannot be successfully treated by any other means than its entire removal. But to be certain that such pain as may have occurred did not proceed from inflammation of the dentine affecting the pulp, the cavity must be examined with great care. In the first place, I carefully wash out the cavity with tepid water, used by means of a syringe, having a tube, curved nearly at right angles with the barrel. This will be found a most valuable adjunct in this and other operations on the teeth. In many cases where the bottom of the cavity can be plainly seen, a small opening into the pulp cavity will be discovered. If this is not apparent, I remove carefully, with a small instrument, the softened bone situated immediately over the most prominent point of the pulp. In these cases it will generally be found so soft that a delicate instrument used gently—and all rough and rude manipulations ought to be carefully avoided in these cases—will push through it. If, however, it should be found to possess considerable consistence, it will be advisable to attempt the operation above alluded to, unless pressure produces so much pain as to put it out of the question.

Inflammation of the pulp may be attributed to two causes, one proceeding from actual contact with some substance or agent which is foreign to it—the other from the irritation caused by a highly inflamed state of the dentine. In both species, pain occurs, but one will, in some cases, subside, and leave the pulp in as healthy condition as any other tissue, after the subsidence of inflammation; the other may, indeed, pass away, but the pulp has previously been deprived of the protection with which nature has furnished it, and which it may not be able to restore, and it is probably liable to a return of the inflammatory condition.

When inflammation exists, characterized by violent pain at the time a case comes under treatment, there will be little question about the condition of the tooth, or the steps to be taken.

I find it extremely difficult, without being too diffuse, to give such full directions to the tyro in the profession as will enable him to treat, without embarrassment, the variety of cases of this kind which will come into his hands—experience alone will enable him to do this—and these hints, I am aware, can be valuable only in assisting him at the outset to avoid some serious difficulties. To the practiced operator, these details will probably be tedious and useless.

After having indicated, to some extent, the means by aid of which to ascertain whether the pulp is in such condition as to render its extirpation necessary, I come now to a closer consideration of the process by which this is accomplished. I have already discussed the relative propriety of its removal with or without the use of arsenic for the purpose of first destroying its vitality, and have declared my decided preference for the latter method.

Even with this invaluable agent for the destruction of the vitality of the pulp, great difficulties have been encountered in making it effectual; and there are many points about the best means of applying it, and the symptoms to which it gives rise, of importance to the novice in its use, and well worthy to be examined in detail.

The form in which arsenic is generally used for this purpose, is that of the arsenious acid, (As Oz,) known also as arsenic, white oxyde of arsenic, white arsenic, ratsbane. A few words in relation to its nature and properties may be useful.

Arsenious acid is sometimes found native, but generally in combination with copper, iron, cobalt, and nickel, as a sulphuret, from which it is obtained by roasting these ores. It is principally obtained for commercial purposes from cobalt, in which it sometimes exist, in the large proportion of fifty or sixty per cent. It is abundantly prepared at Joachmil, in Bohemia, from arsenical pyrites, and arsenical cobalt, which are roasted in reverberatory furnaces, and the vapors condensed in a long flue, or series of chambers, the contents of which, submitted to a second sublimation, affords the *white arsenic* of commerce.—(*Brande.*) The commercial arsenic is white, transparent, and glossy when fresh, becoming milky and opaque by exposure; so that the two states are observed on breaking a mass, which is opaque on the exterior. As it is sold in powder in the shops, it is often adulterated, and it is always advisable to purchase it in the lump. If wanted perfectly pure, it may be obtained by pulverizing the commercial article, and digesting it for some hours in aqua ammonia, at a temperature of 160° or 170°, frequently shaking the vessel; the clear, warm solution is then poured off, and in cooling it, deposits octohedral crystals of the acid, pure and free from ammonia.—(*Berzelius.*) Arsenious acid is soluble in water, in acids, and oils. The opaque is more soluble than that which is transparent. It has been ascertained that 0.96 of the transparent, and 1.25 of the vitreous, dissolved in 100 parts of water at 60°; at 212° 9.68 of the former, and 11.47 of the latter dissolves, and when these solutions are cooled down to 60°, 1.78 of the vitreous, and 2.9 of the opaque are retained.—*Guibourt.*

On other authority, it is stated, that the transparent variety is much more soluble in boiling water than the opaque.—*(U. S. Dispensatory.)* Used internally, arsenious acid acts as an alterative and febrifuge. It is exhibited in doses of a sixteenth to an eighth of a grain. Applied externally it acts as a caustic.

Besides the arsenious acid, arsenic in another form is used; as it is found combined with cobalt in the cobalt ore. Cobalt is generally of a dark, or reddish gray color. It sometimes contains as much as 55 per cent. of arsenic.—*(Ure.)* It is not soluble in water, or oils, at any temperature.

I have used cobalt a great deal for the purpose of destroying the pulp, for some time past; I found that it possessed some advantages over arsenious acid when employed in the usual way; but a new method of using the arsenious acid, which I shall presently describe, gives this agent a decided preference over cobalt in consequence of its more easy solubility, and more rapid action. Indeed, on reflection, upon a suggestion made by Dr. White, I do not see how, theoretically at least, cobalt should be preferred to the arsenious acid; for if the pulp is to be destroyed, the more quickly this is accomplished the better. But for the purpose of removing that excessive sensibility of the dentine, described in a former number, this agent is invaluable; and I cannot refrain from taking this opportunity again to urge a trial of it, for this purpose, upon the profession. I have now been using it for nearly two years with the most satisfactory results.

The preparation of arsenious acid generally used for the purpose in question is, equal parts of arsenious acid and sulphate of morphia, rubbed up in creosote, to the consistence of a thin paste. As creosote is so disagreeable to many persons, it has been proposed to substitute for it some of the essential oils.

A writer in the "Journal" has suggested oil of origanum. I have not tried any of these, but think the suggestion a good one.

This compound is used instead of the simple arsenious acid, in order to prevent the pain caused by the destructive action of the acid. It has been suggested by Dr. White that the creosote has no agency in diminishing the pain caused by the action of the arsenic, except in dissolving the arsenic more perfectly, bringing its particles more certainly and rapidly into contact with the pulp, and thus so quickly destroying its vitality as to prevent irritation. The morphine, in the preparation, he supposes to be the agent which prevents the pain, which would otherwise be produced. I do not, however, agree with him in this view of the case.

About the twentieth, or even thirtieth part of a grain of arsenious acid, it is generally agreed, is sufficient to effect the destruction of the vitality of the body of the pulp. This is applied by touching to the arsenical paste, a small piece of raw cotton or lint, and laying it in the cavity, against the part of the cavity nearest the pulp. If cobalt is used, it will be found simply sufficient to saturate the cotton with creosote and touch one side of it to the powdered ore.

A very nice and important step in the process, is properly to secure in the cavity the preparation employed. As the exposed pulp is often exquisitely sensitive, it will not bear the slightest pressure, and it was at first found quite difficult to confine the preparation in the cavities of some teeth without making so much pressure upon the pulp as to render the process exceedingly painful. Several methods have been devised to escape this difficulty. Dr. Maynard covers the preparation with a lead cap, and then fills the cavity of decay with wax, into which a quantity of cotton is worked. The lead cap, it is obvious, so protects the pulp, that enough pressure can readily be used to make the wax sufficiently firm to remain as long as desired. Dr. White suggests passing a ligature around the tooth where the cavity is on a lateral surface, and when difficulty is experienced in securing the cotton placed over the preparation. In some cases, this method is invaluable. The means I have generally used, and which in the large majority of cases I find perfectly effectual, is to make an alcoholic solution of gum sandarac, and saturate with it a piece of cotton sufficient to fill the cavity over the preparation. When the cavity is deep enough, and sufficiently well shaped to hold a filling of any kind, cotton prepared in this way is very easy to use for the purpose, it will effectually exclude moisture, prevent the escape of a particle of the preparation, and remain any desired length of time. The saliva, on coming into contact with it, immediately combines with the sandarac, the alcohol evaporates, and a firm filling is quickly produced.

I do not regard the complete exposure of the pulp, by the removal of all the decomposed bone which may cover it, as a necessary preliminary to the application of the arsenic. On the contrary, much pain to the patient is often avoided by applying the preparation without first removing any bone by which the pulp may still be covered. This practice, I am aware, conflicts with what appears to be correct theory in relation to the matter; for it might be supposed, as has been suggested by Dr. White, in the articles before alluded to, that the action of the arsenic upon the pulp would produce irritation and a consequent flow of blood to the part, so that the usual consequences of the inflammation of any tissue in a confined bony cavity would follow, and great pain occur. Experience, however, my experience, at least, shows that this is not the case, and it will often be found, where the patient is suffering violent pain, and the affected pulp is still not fully exposed, that the application of either of the arsenical preparations, as above directed, will, instead of augmenting the pain, give almost immediate relief. In most cases, indeed, this action of the preparations of arsenic, when combined with creosote, seems to give rise to but little, if any, irritation.

Whenever, therefore, I determine upon the performance of this operation, I merely syringe out the cavity of the affected tooth with tepid water, dry it, and immediately, without any farther preparation, except, in some cases, to cut away the softened bone near the opening of the cavity, so that it will more readily retain what is placed in it—make my application. After the lapse of twenty-four hours the pulp may be fully exposed with but little pain.

There is some controversy about the length of time the arsenic should be allowed to remain for the purpose in question, and also with regard to the propriety of a repetition of its application. The common practice is to apply it in the manner above described and to allow it to remain from twelve to forty-eight hours, and there seems to be a great and general reluctance to apply it more than once. It will be found, however—at least such has been my own experience—that the pain attendant upon the removal of the pulp is not avoided, in any great degree, by the use of arsenic in the way just mentioned. When the arsenic is removed, after having remained only the length of time just indicated, the body of the pulp will be found entirely deprived of vitality, and of course sensibility, and can be removed with but little inconvenience to the patient, but farther than this, the effect is rather an exaltation of the sensibility of the portion still remaining in the fang or fangs; and the removal of this part of the pulp is, in almost every case, exceedingly painful. To destroy the vitality of this part it has been proposed to repeat the application of arsenic; but this has been objected to on the ground that bad consequences were likely to result from it by its passage through the foramen to the investing membrane. In a preceding number of these papers I have examined this question. I have had no hesitation in applying the arsenic repeatedly for this purpose, and have never seen any injury result from it. The same has been stated to me by Dr. Maynard to be his own practice and experience. It has been proposed to substitute for the arsenic some caustic more rapid in action and less diffuse in its effects; I have carefully tried for the purpose, caustic potassa, nitrate of silver, chloride of zinc, and other similar agents without satisfactory results.

The difficulty with all these agents, arsenic included, even if no fears of injurious results from its use were entertained, lies in the impossibility, by any methods known to me, of bringing them into contact with the parts of which it is desirable to destroy the vitality after the body of the pulp is removed. Till lately this has been an extremely perplexing point to me, and I will mention briefly the various expedients which have been resorted to in order to overcome the difficulties of the case. Dr. Maynard mentions two methods adopted by him, viz. to apply the preparation by means of a piece of thread, the end of which he dipped into it so as to take up the desired quantity and passed into the fang; and to take up some of the preparation upon a fine instrument and passing it to the end of the fang withdraw it, leaving a small quantity of the arsenic behind. I have modified these methods by taking a fine instrument, warming the point and touching it to gutta percha so as to take up a very small quantity of the gum. This was touched to the arsenical preparation and thus armed was passed into the fang to the extremity and allowed to remain twenty-four hours. Other expedients of the same nature were tried, and although many of them promised well, did not meet my expectations when applied to practice.

In reflecting upon the matter, it was plain to me that, although considerable inflammation of the portion of the pulp not destroyed by the arsenic, occurred, and even extended to the peridental membrane, that no particles of arsenic reached these parts; for if the inflammation in question were caused by the presence of particles of arsenic, the death of the parts would speedily follow, but as these parts retained their vitality for weeks and months afterward, it was fair to infer that they could not have been reached by any particles of the preparation. But it appeared to me equally plain that if a sufficient quantity of the arsenic were used, the pulp itself would answer as a vehicle to convey it not only to the extreme parts of the fang but, if allowed to remain long enough, to the peridental membrane; for as the surface to which the arsenic is applied in the first instance is destroyed, new portions, combined as it is with so subtle a fluid as creosote would readily pass by capillary attraction through the dead to the living parts. It appeared to me then a great mistake to remove the pulp, as it is deprived of vitality, for this, if my view is correct, is taking away the best means of bringing the arsenic into contact with the part of the pulp most difficult to reach.

Reasoning somewhat in this way, I determined to allow my preparation to remain a longer time, and after some experiments proposed about a week, when cobalt and creosote were used. I found this plan much more effectual than any other which, up to that time, I had tried, and warmly recommended it to the profession. It was, however, with reason, urged against this method that it was impossible to tell with exactness the extent of the action of the arsenic in any given time, and there was danger of allowing it to remain so long as to pass through the foramen and thus destroy the vitality of the membranes within the alveolus. It was necessary, therefore, in order to be on the safe side, to allow its action to stop short of the foramen, and leave a portion of the still vital pulp to be taken away. For this reason, although a great improvement upon the old method, it was so far defective, and the termination of the operation was still more or less painful.

But this step led me to a method which is free from any of the objections and defects which I found in that just described, answering effectually the desired purpose, and placing in the hands of the operator the means of determining, with exactness, the progress of the action of the arsenic applied, and of stopping it at the desired point. This method removes from the process its most perplexing feature to the operator and the most repulsive to the patient.

I have now discarded the cobalt and again taken the arsenious acid, because it is soluble in the creosote, and because I desire more rapid action. It will be perceived by those who have followed me thus far, that I have arrived at these conclusions since the last number of these papers was written.

I have just said that I am satisfied that if a sufficient quantity of arsenic were used and allowed to remain long enough, it would travel along the pulp and effect its entire destruction. More, probably, would be required for the purpose than is generally used, for as it combines with the pulp, as it destroys its vitality and becomes inert, there must, of course, be a sufficient quantity to act upon the whole. If it were possible to know the exact amount required, in every case, to effect this object, a great deal of trouble would be saved—but this is, of course, in the nature of things, impossible. I therefore apply the arsenious acid in the usual quantity and in the usual manner.

After the lapse of twenty-four hours, knowing that the vitality of the whole of the pulp is not destroyed, and not knowing whether the minute quantity of arsenic employed has not exhausted itself, I renew the application. On the next day, I remove it, and pass a very fine instrument, prepared for the purpose, into the fang, till a sensitive point is reached, making a mark upon the instrument to indicate the depth to which it has gone. I apply the arsenic again, and the next day examine with the same instrument again, and so on, till the vitality of the pulp is completely destroyed to the end of the fang. The action of the arsenic is not so rapid as to give any ground for apprehension of injury, and sufficiently so to effect the desired object in about a week In this manner I have succeeded in destroying effectually the pulp, without observing a trace of inflammation about the tooth.

If the tooth under treatment has several fangs, the instrument used may be passed into each, and as the vitality of the pulp in each fang is ascertained to be destroyed, its connexion with the main body of the pulp may be cut off.

I have had a good deal of experience in this practice of destroying the nerve, and I do not hesitate now, from the trials I have made of the process proposed, to declare that it accomplishes an object which I have not yet been able by any other means to effect—certainty of results.

After the vitality of the pulp is completely destroyed, its removal, in some cases at least, is not very difficult. In the incisor and canine teeth, it is quite easy, but more difficult of course, in the bicuspis and molar teeth. This part of the operation demands some special attention.

The first thing to be considered is, the instruments necessary for the purpose. It is, of course, understood, that in most cases, all which has to be done, is to remove from the fang or fangs of the tooth under treatment, a comparatively soft substance unlike the decayed or decaying bone of the original cavity of decay, and not requiring so high a temper as ordinary excavators. But in regard to instruments intended for this purpose there are several particulars to be observed. They must be small enough to pass into the canal of the fang, to the very extremity—they must have some elasticity, enough to take the slight curves presented by many fangs coming under treatment, and that formed by the relative position of the fang to the opening from the external cavity—they must also possess sufficient strength and toughness to prevent them from breaking when used for the purpose in question—and must be given such form as will enable them to seize hold of the pulp and draw it out. The best material for these instruments which I have yet found, is fine steel, well annealed. The best steel for the purpose, of which I have any knowledge, is piano wire.

Steel is annealed by being heated away from contact with the oxygen of the atmosphere, and kept from contact with it until it becomes cold. A convenient method of annealing steel wire for the purpose in question, is to cut it into proper lengths, and put it into a piece of gun barrel, in each end of which has been fitted an iron plug to render it air-tight. It should be heated to redness and allowed to remain on the hearth where it is heated till it becomes cold. The more slowly this operation is performed the more uniform and soft will be the steel.

After annealing the wire, cut into the proper lengths for the instruments, it is fitted into suitable sockets. For this purpose I generally use steel wire with a socket drilled in it, into which the wire for the instrument is cemented. But any convenient form which may suggest itself to the operator, will, of course, answer. It will be found convenient to have a considerable number so that there may be at hand, instruments of a variety of sizes, thus saving the necessity of frequently changing them.

When securely fastened into the sockets they may be filed down to the required size. It is advisable for those who have never practiced this operation to prepare teeth of the several classes, and make the instruments of such size that they will readily pass into the smallest fangs. In order to obtain the most desirable form, and to preserve as much body as possible, to the instrument, it has been suggested, (by Dr. White,) that they should be square, except in cases where it is necessary that they should correspond with the form of the fang. After they are reduced to the desired size, they should be well burnished, (a suggestion of Dr. Maynard ;) this will give them a slight temper, and increase the little elasticity they have acquired in the process of filing. There should now be cut upon them with a sharp knife, small beards, looking toward the handle of the instrument. Care must be observed, too, that the points of these little instruments should be made sharp, so that they will pass alongside of the pulp in the fang, and not push it upward in the attempt to take it away. The use of these instruments is too obvious to need any elaborate description ; they pass readily into the fang and will draw out the pulp as they are withdrawn.

Sometimes the fang will require enlarging, and this may be done with a very simple excavator, contrived by Dr. Maynard. It is formed somewhat like a hoe, but with the cutting edge at a more acute angle with the handle. It is made, of course, of various sizes, to suit any case in hand. When used, it is passed into the fang, and pressed slightly to the side toward which the cutting edge is turned and withdrawn—it cuts as it is withdrawn, and brings away with it the chips it makes. It is important that it should be made at a proper angle, for if it varies from this it will either not cut at all, or merely scrape the bone. If properly made and sharpened it will cut quite rapidly. This little instrument is exceedingly useful, too, for enlarging the opening of the pulp cavity before the vitality of the body of the pulp is completely destroyed ; for this purpose it is passed gently into the pulp cavity and in withdrawing it the edges are gradually cut away.

Several little matters are to be noted at this place. It is not always so easy as it may appear to the inexperienced to remove the pulp from the fangs. It is not always sufficient to pass the instrument prepared into the fang to bring out the pulp with it on the first trial. The pulp is often toughened by combining with the arsenic or cobalt, holds firmly and resists repeated attempts to take it away. If any vitality remains, this part of the operation becomes exceedingly perplexing and painful to the patient; but I hope the method which I have suggested will obviate this difficulty. Not unfrequently the pulp will be found partially ossified. These ossifications of the pulp sometimes present themselves in the form of spiculæ in the fangs; but I have sometimes found the whole body of the pulp completely ossified up to the openings of the fangs. These ossific deposits will be found free, but often it will be necessary very much to enlarge the opening to the pulp cavity to get them out of the way.

If, by accident, the instrument used should break—and this is an accident which careful handling should make very rare—it will sometimes be found difficult, and it may be impossible, to remove. If not jammed in the fang so as to be immovable, it may, in many cases, be withdrawn by rendering a small instrument magnetic, and passing gently up till it comes into contact with the fragment to be removed. The use of a magnetized instrument was suggested some time ago by the late Dr. John Harris, for a similar purpose. Once or twice during my practice I have found it impossible to remove the broken fragment of the instrument from the fang, and was obliged to fill without regard to it. I have observed no unfavorable results in these cases which I could attribute to this cause.

There is another important matter which may be appropriately considered here, although it has a more important bearing upon the perfect filling of the fang after the pulp is removed, viz. the best means of gaining access to the fangs of the teeth under treatment. This, at times, requires the exercise of considerable ingenuity, and unless the proper means are resorted to, in many cases the operation will necessarily be imperfect and ineffective. What I mean, will be more clearly understood, by taking, for example, the case of an inferior molar tooth, decayed to the pulp on the posterior proximate side. It is obvious that it will be difficult to reach the opening of the anterior fang from this cavity, and, indeed, it will also be found quite difficult to work readily at the posterior fang, unless a considerable portion of the tooth toward the anterior proximate surface is cut away. This opens a novel and interesting feature of this operation, and the question now comes, what is the best method of gaining access to these fangs. Cutting away the crown of the tooth, as in that chosen for illustration, so as to reach the fangs, has two objections: it is a very laborious process, and takes away a large portion of the tooth which would be useful for mastication, and although this portion may be restored by the filling, it also involves a great deal of labor, consumption of material, and necessarily adds to the cost of the operation. A much more easy and feasible plan is to make an opening somewhere through the sound bone, and as there is now a choice of location it will be well to consider and ascertain what part is more convenient for the purpose. In order to make this part of my subject plain, I shall take up for consideration each class of the teeth separately. I feel bound to say here, that I am indebted to Dr. Maynard for suggestions in relation to this matter, which have been of great value to me.

*Incisor Teeth.*—When the pulp is exposed on one of the lateral or proximate surfaces of these teeth, if the cavity is not very large it will be best to make the opening, for operating upon the pulp, on the lingual surface just below the most convex point, particularly in case of the central incisors. Through this opening the necessary instruments may be passed almost vertically into the canal of the fang. It is objectionable to cut down toward the lingual surface, if the cavity of decay is small, because it so far takes away from the strength of the tooth. It will of course be understood that when force is applied in a vertical direction, even with the most delicate instruments, it will be at a great advantage over its application out of this line, and the filling of the fang can doubtless be made much more perfect.

The inferior incisor teeth cannot be treated in this way—it would be more difficult to reach the pulp cavities of these teeth from the lingual than from the proximate surfaces. All that can be done is to cut away the labial wall of the cavity till the openings of the fang are fully exposed. As these teeth are generally less conspicuous than the superior incisors this treatment is admissible.

*Cuspidati.*—There are two reasons why it will generally be found advisable when it is desirable to reach the pulp to use the cavity of decay when it occurs on the proximate surfaces of these teeth for this purpose, rather than to make a new opening on the lingual surface. In the first place, these teeth are thicker across from the labial to the lingual surface than the incisor teeth, and can, without injury, bear greater loss of substance, and for this reason the pulp cavity is less easily reached from the point indicated above than in the incisor teeth.

*Bicuspids.*—The manner in which these teeth most commonly decay, renders any particular directions unnecessary. From the cavities of decay it will generally be found easy enough to reach the pulp cavity. If, however, the pulp is exposed from decay on the posterior proximate surface, and the anterior surface is untouched, it may be well to say that the pulp cavity can be most easily reached by cutting down the cavity of decay toward the anterior surface.

*Molar Teeth.*—It is with these teeth that the greatest difficulties are encountered in the treatment of the pulp. The variety of places in which they may be decayed, their position in the mouth, and the number of their fangs, all serve to make a very considerable exercise of ingenuity necessary. I can only hope to afford some help by indicating, in as full detail as seems useful, the general plan resorted to to overcome these difficulties.

The *Superior Molar Teeth*, for some reasons, are more troublesome to treat in this way than those of the lower jaw, unless the caries has fully opened the pulp cavity and exposed the entrance to the fangs. I shall examine these teeth with reference to this object, as they are affected by caries, in most of the situations where it is likely to affect them. *Anterior proximate surface.*—From this point, if the caries is extensive, all the fangs may be reached, by cutting down the pulp cavity toward the center of the crown. If the caries is slight in extent, an opening made near the gum on the labial surface will make it easy to reach the buccal fangs. The palatine fang in such cases may be reached by cutting down the cavity of decay toward the point formed by the lingual and anterior proximate surfaces. *Posterior lateral surface.*—Through an opening at this point but little more can be done than to make the first application of the arsenic. I have reached the pulp cavity in such cases by making an opening at the center of the crown and cutting down to the pulp cavity. This will be found a laborious operation, but I do not see how it can be avoided, unless openings are made both in the lingual and labial surfaces, or a very large opening on the labial surface. *Lingual surface.*—In this case the palatine fang only is accessible; to meet the difficulty, an opening must be made on the labial surface as before directed. *Labial surface.*—From this point as it is more accessible, the palatine fang, if the decay is large, may be reached, at an obtuse angle.

It will, perhaps, be understood that these additional openings into the pulp cavity cannot be attempted until the vitality of at least the body of the pulp is destroyed. This is, of course, done by making the arsenical application through the original carious opening. It will, however, be necessary to gain easy access to the fangs before the entire pulp can be effectually deprived of vitality and removed.

*Inferior Molars.*—These teeth, I think, will generally be found less difficult to treat in this way than those of the upper jaw, with the exception of the trouble encountered with the saliva; there is also a difficulty arising from the form of the fangs, which, it is known, are generally flattened laterally, slightly enlarged both toward the tongue and cheeks, forming, in each fang, two canals with a narrow fissure between them. When these teeth are decayed to the pulp cavity on the crown, it is only necessary to cut down into the cavity and open a free way to the fangs. If it is on the anterior surface it will be sufficient to cut down the roof formed by the bone of the crown, so as to reach the posterior fang. If the caries is in the posterior proximate surface, it will be best to make an opening through the sound bone on the labial surface near the gum, and from this place enter both fangs.

The *dentes sapientiæ* of the lower jaw are not generally more difficult to fill than the first and second molars. If decayed upon the posterior surface it will be best to cut away the crown so as fully to expose the fangs; as these teeth are generally smaller than the other molar teeth this will not be found so laborious, indeed I have usually found when the pulp is exposed from caries at this point, that so much of the bone is decomposed as to allow of easy access to the pulp cavity. When the caries affects the crown no difficulty presents itself. The upper wisdom teeth are more difficult generally to treat than the lower ones, particularly those of the left side, as they are not so easy of access.

I find difficulty in describing minutely operations like these, in consequence of the want of a dental nomenclature. Until every important part of each tooth receives its appropriate name this will continue to be difficult. Good service would be done to the profession by the careful preparation of such a work.

There is a point to be considered here of considerable importance, it is this: how is it to be ascertained with certainty that the pulp is entirely destroyed? At first sight this may appear quite an unimportant inquiry, for it will be supposed that as pain is an indication of vitality, when no pain is caused by the use of instruments for the removal of the pulp, it alone will be sufficient assurance that there is no vitality left. But the difficulty is in this: the canal passing from the pulp to the alveolar cavity does not always terminate abruptly near the extremity of the fang, leaving a mere capillary foramen through the fang; there is often a gradual and regular narrowing of this canal till it reaches the alveolus, and not unfrequently the foramen is sufficiently large to permit the passage through it of the delicate instruments used in this operation. This will be shown by passing such instruments through the pulp cavity and fangs of extracted teeth; it will be found in doing this that the finer instruments will readily pass through many of them, even when the foramen is not very perceptible to the naked eye.

This, it will be perceived, is a nice point—it is certainly desirable, at least, if not necessary, as I have endeavored to show, to remove entirely the pulp from the canal; but how to accomplish this, in all cases, without running risk of wounding or otherwise injuring the parts exterior to the fangs, and setting up inflammation here which it will be difficult to subdue, is a question well worthy of attentive consideration. In many of the teeth the canal terminates somewhat abruptly, near the extremity of the fang, and the instrument used will be stopped at this point. Sometimes the difference between the diameter of the canal at this place and that of the foramen is so slight that the instrument used, although stopped at first, may be passed through the foramen if a little force is employed. In these cases the obstruction to the passage of the instrument indicates the point at which the canal terminates. When the pulp has been removed up to this point, even if some sensibility still remains, it is not necessary to go farther, and to this place the gold is to be carried in filling. It will, of course, be understood that the depth to which the instrument used has passed into the fang, must give assurance that the obstruction is near the extremity.

But it will often be found exceedingly difficult to determine with certainty whether a sensitive point touched by the instrument is a portion of the pulp still remaining in the fang or the part exterior to this. I am aware now that, sometimes, in the earlier part of my practice, I have inflicted severe and unnecessary pain in repeated attempts to remove what I supposed to be some remaining portion of the pulp, when I was afterwards satisfied, that at every attempt thus made, my instrument passed entirely through the fang. This is more to be apprehended in the treatment of teeth in which the vitality of the pulp has been for sometime destroyed; and particularly so if they have been the seat of alveolar abscess. In such cases the canal of the fang and foramen are very often so much enlarged by absorption, as to admit the passage of quite a large sized instrument. In filling these fangs, Dr. Maynard tells me he is in the habit of measuring the length of the canal by using a fine instrument with the point bent at a right angle; this he passes up the root till it snaps over one edge of the foramen at the extremity of the fang, he then marks on his instrument the depth to which it has gone, and with another suitable instrument carries up the gold exactly to the end of the fang.

In cases where the pulp is not dead, and where there is no enlargement of the foramen, and there is but slight difference between its diameter and that of the canal, it is extremely difficult to know the proper point at which to stop, and the only means of which I know anything, and by which I am guided myself is merely an approximation. I judge from the depth my instrument has passed into the fang of its length, by comparing it with the average length of the fangs of teeth of the same class. It will be well for young practitioners who have not in their minds some adequate idea of the average length of the fangs of the teeth, to keep by them one or more of each class, and compare the length of these fangs with those under treatment. Although this is but an approximation to the desired knowledge, still it furnishes the means of operating with much more certainty than could otherwise be done, for although there is a difference between the length of the fangs of the teeth of the same class of different individuals, this difference is not so considerable, generally, as to make this method of ascertaining their length, entirely unavailable. And even if, occasionally, the length of the fang under treatment should be somewhat longer than it is supposed to be, it will be better to leave a minute portion of the pulp remaining in the extremity of the fang, than to run the risk of wounding the parts exterior to it.

During the process of removing the pulp, or after it is completed, some peridental inflammation is very likely to occur. I have found it much less apt to present itself since using the arsenious preparation in the manner I have described; and I am inclined to believe, from what I have observed, that a greater amount of peridental inflammation is likely to occur from rough attempts to remove the pulp without regard to the pain inflicted than from the effects of the arsenious acid. I have always, indeed, observed, that in those cases where most pain attended the removal of the pulp, most peridental inflammation followed. This inflammation of the membranes of the socket, for doubtless the living membrane of the alveolus from its contiguity is involved in the trouble, is indicated by tenderness to pressure, and this may vary in degree from slight inconvenience in mastication to severe pain from simple contact with the teeth of the opposite jaw, and even when no such cause as this gives rise to it, to severe and continued pain. Generally when the removal of the pulp is completed some slight inflammation will be present, and it will always be found advisable to wait till this subsides before proceeding with the operation. In most cases the inflammation which occurs will pass away spontaneously, but it may require treatment. It is always best to attend to it without delay, for although in the beginning simple measures will generally effectually arrest it, after it reaches a certain point it will in spite of all treatment run on to suppuration. It will generally be found that chloroform applied along the course of the fang on a small piece of cotton saturated with it, acting as a derivative and probably as a sedative, will effect all that is desired; but if this will not answer, the application of leeches to the part will generally have the desired effect. In using the chloroform, it is necessary to caution the patient not to use it too freely.

In one case in my hands, a lady of delicate habit declared that she was rendered insensible and quite sick afterward by the use of a very small quantity in the manner directed. I could scarcely credit the statement at the time—concluding that the quantity used was much greater than the lady was aware of; but Dr. White has since informed me that he observed in his office, if I am not mistaken, a similar result from a very small quantity of the chloroform. I have made frequent use of it, however, many times with very happy results, and never in any other case met with such consequences.

Sometimes this inflammation of the membranes will continue for a considerable time, and it is best, except when the case is urgent, to wait, using, in the mean time, all appropriate remedies till this subsides, before filling the fang. I do not know that a disregard of this injunction would absolutely prevent the success of the operation, but it is certain that increased irritation, caused by the force necessarily used in filling would have a tendency to augment the inflammation of the parts, when they were already in an irritated and irritable condition.

But there is another reason why some interval should elapse after the pulp is removed before the fang is filled. Especially is this the case if the removal of the pulp has been rapidly accomplished. A quantity of blood has been regularly brought to the pulp, and when this part of the tooth is in healthy condition has been regularly returned to the system. The vessels bringing the blood into the fang are cut off, and there is an excess of blood which must be diverted to some other use, which is done by the adaptation of the surrounding vessels. Before this is perfectly accomplished, and before the excised vessels are perfectly healed, there will be a greater or less exudation of coagulable lymph, which, if not allowed to escape freely, must react upon the parts from which it has been thrown off, and thus cause trouble.

Nothing now remains to be done, after allowing sufficient time to elapse, with the assistance of proper remedies to get rid of any inflammation occurring about the parts, but to complete the operation by filling the fangs and the cavity or cavities in the crown of the tooth under treatment. This is a nice and delicate part of the process, often requiring the exercise of considerable ingenuity, and always demanding steady and patient manipulation on the part of the operator. This will form the subject of the next and concluding number of these papers.

